# Effect of remote ischemic postconditioning in inflammatory changes of the lung parenchyma of rats submitted to ischemia and reperfusion

**DOI:** 10.5935/1678-9741.20150005

**Published:** 2015

**Authors:** Rafael Cantero Dorsa, José Carlos Dorsa Vieira Pontes, Andréia Conceição Brochado Antoniolli, Guilherme Viotto Rodrigues da Silva, Ricardo Adala Benfatti, Carlos Henrique Marques dos Santos, Elenir Rose Cury Pontes, José Anderson Souza Goldiano

**Affiliations:** 1 Federal University of Mato Grosso do Sul (UFMS), Campo Grande, MS, Brazil.; 2 General Director of the Nucleus of the University Hospital of the Federal University of Mato Grosso do Sul (UFMS), Campo Grande, MS, Brazil.; 3 Assistant Professor in Cardiovascular Surgery at the Federal University of Mato Grosso do Sul (UFMS), Campo Grande, MS, Brazil.; 4 Specialist in Cardiopulmonary Bypass of the Federal University of Mato Grosso do Sul (UFMS), Campo Grande, MS, Brazil.

**Keywords:** Ischemia, Reperfusion, Ischemic Postconditioning, Lung Injury

## Abstract

**Objective:**

To assess the effects of postconditioning remote in ischemia-reperfusion injury in rat lungs.

**Methods:**

Wistar rats (n=24) divided into 3 groups: GA (I/R) n=8, GB (R-Po) n=8, CG (control) n=8, underwent ischemia for 30 minutes artery occlusion abdominal aorta, followed by reperfusion for 60 minutes. Resected lungs and performed histological analysis and classification of morphological findings in accordance with the degree of tissue injury. Statistical analysis of the mean rating of the degree of tissue injury.

**Results:**

GA (3.6), GB (1.3) and CG (1.0). (GA GB X *P*<0.05).

**Conclusion:**

The remote postconditioning was able to minimize the inflammatory lesion of the lung parenchyma of rats undergoing ischemia and reperfusion process.

## Abstract (pt)

**Objetivo:**

Avaliar os efeitos do pós-condicionamento remoto no fenômeno de isquemia e reperfusão nos pulmões de ratos.

**Métodos:**

Ratos Wistar (n=24) divididos em 3 grupos: GA (I/R) n=8, GB pós-condicionamento remoto n=8, GC (controle) n=8, submetidos à isquemia de 30 minutos por oclusão da artéria aorta abdominal, seguida de reperfusão de 60 minutos. Ressecados os pulmões e realizada a análise histológica e classificação dos achados morfológicos de acordo com o grau de lesão tecidual. Análise estatística das médias da classificação do grau de lesão tecidual.

**Resultados:**

GA (3,6); GB (1,3) e GC (1,0). (GA X GB *P*<0,05).

**Conclusão:**

O pós-condicionamento remoto foi capaz de minimizar a lesão inflamatória do parênquima pulmonar de ratos submetidos ao processo de isquemia e reperfusão.

**Table:** 

**Abbreviations, acronyms & symbols**	
AMP	Adenosine monophosphate
ATP	Adenosine triphosphate
COBEA	Brazilian College of Animal Experimentation
IPoC	Ischemic postconditioning
IPreC	Ischemic preconditioning
R-PosC	Remote postconditioning
TROS	Toxic reactive oxygen species

## INTRODUCTION

Ischemia is a condition of interruption of the supply of oxygen and nutrients for a given area during a period, due to deficiency of arterial blood supply, and is known to be cause of dysfunction and subsequent death of tissues in many clinical situations, e.g., in acute myocardial infarction, pulmonary infarction, mesenteric infarction, ischemic stroke and limb ischemia^[1]^.

Reperfusion of ischemic organ is essential to its viability and functional recovery. However, the arrival of the blood will cause a number of lesions that were called ischemia-reperfusion injury, this term refers to a variety of changes at the time of restoration of blood flow and the impairment of function until the cell death^[2]^.

In addition to the lesions which occur in the tissues which pass through the ischemia and reperfusion process, it is known that distant organs also suffer damage caused by this process^[1]^.

A classic example is the ischemic preconditioning (IPrC), which initially proved to be effective in treating the target organ of the ischemic process, and later studies have also shown its protective effect at a distance, the called remote ischemic preconditioning^[3]^.

In the last decade, there have been studies demonstrating the efficacy of ischemic post-conditioning in various organs when subjected to ischemia and reperfusion^[4]^. Clearly, then came the questions about its protective effect also at a distance, the remote ischemic postconditioning (R-IPo).

In 2005, Kerendi et al.^[5]^ were the first to introduce the R-IPo strategy, which consisted of brief period of ischemia and reperfusion, which reduced the size of the infarcted area in the heart of rats.

Despite this and other studies^[6,7]^ that assessed the effect of R-IPosC, the literature is still scarce on this topic, especially on the effect in the lung parenchyma, which is considered of great interest, given the importance of the integrity of lungs in patients who undergo ischemia and reperfusion processes for their establishment.

The aim of this study is to assess the ability of R-IPo to minimize injury in the process of ischemia and reperfusion in the lungs of rats subjected to ischemia and systemic reperfusion.

## METHODS

This study was approved by the Research Ethics Committee of the Federal University of Mato Grosso do Sul under No. 296 ratified by the Ethics Committee on the use of animals/CEUA/UFMS on September 12, 2011.

24 Wistar adult male rats (*Rattus norvegicus albinos*, Rodentia, Mammalia) were used, weighing between 250-350 grams, with an average of 310 grams, raised in the conventional-controlled vivarium of Mato Grosso do Sul Federal University. The animals were kept in controlled conditions of light (light cycle from 7 am to 19 pm), temperature (22ºC-24ºC) and receiving standardized ration and water *ad libitum*, attending to the observations advocated by the Brazilian College of Animal Experimentation (COBEA).

The animals were divided into three groups:

Group A - Ischemia and reperfusion (I/R): Eight rats subjected to ischemia for 30 minutes by occlusion of the abdominal aorta just below the diaphragm, with vascular clamp (Clamp Bulldog De Bakey, gentle, curve, 5cm, EDLO^®^) followed by reperfusion for 60 minutes for removal of the clamp.

Group B - Remote ischemic postconditioning (R-IPo): Eight rats subjected to ischemia and reperfusion procedure as described above. Among ischemia and reperfusion, there was R-IPo reperfusion for three cycles (two minutes each) interleaved with three cycles of ischemia (two minutes each), respectively, by removal and repositioning of the clamp. Group C - Control: Eight rats subjected to aortic dissection and handling in a manner similar to the groups A and B, but without applying vascular clamp.

The animals were weighed on an electronic precision balance (Callmex^®^ - model Q510) and anesthetized with an intramuscular injection in the right posterior limb, of solution of 2:1 of Ketamine hydrochloride (Cetamin^®^), 50 mg/ml, and Xylazine hydrochloride (Xilazin^®^), 20 mg/ml, respectively, at a dose of 8 mg/100 g associated with 1 mg/100 g.

**Fig. 1:**
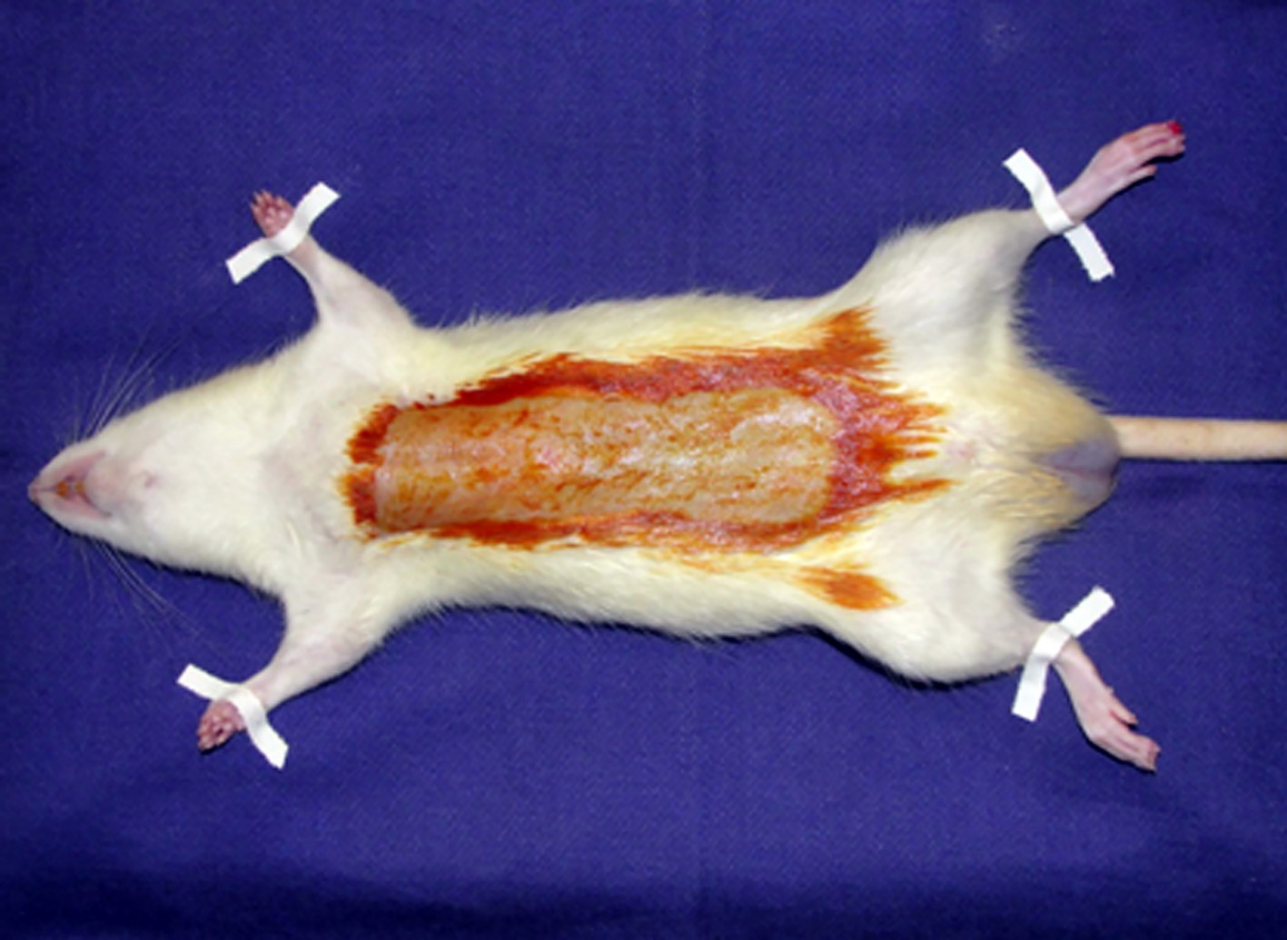
Rat positioned on the operating table with four limbs abducted and antisepsis with iodine performed.

After verified the anesthesia, the rats underwent abdominal trichotomy, positioned at the operating table with four abducted limbs and performed topical antisepsis (Figure 1). After placement of surgical fields, the rats underwent median laparotomy of four centimeters, dissection and identification of the abdominal aorta.

**Fig. 2:**
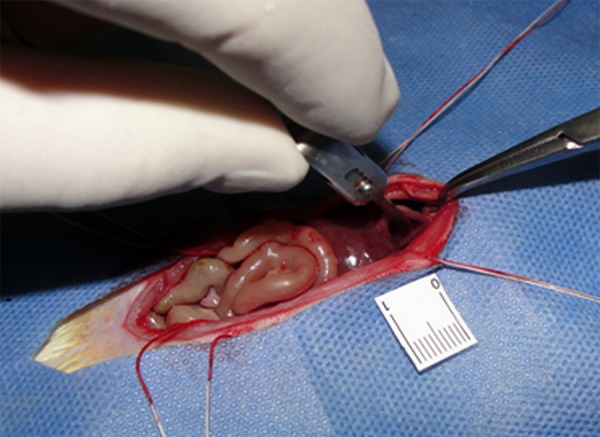
Clamp in the abdominal aorta.

In group A, the abdominal aortic artery was occluded with atraumatic vascular clamp which remained for thirty minutes (ischemic phase) (Figure 2). In all three groups the wound was covered with gauze moistened with saline solution at 0.9%. After the stage of ischemia, vascular clamp was removed, beginning the reperfusion phase, lasting 60 minutes. In all three groups, the surgical wound was closed during the reperfusion sutured by simple running suture using 3-0 mononylon.

In group B, ischemia phase (30 minutes) and reperfusion were performed (60 minutes). When preceding the reperfusion the PosC was performed through three cycles of reperfusion (removal of atraumatic vascular clamp from the abdominal aortic artery) lasting two minutes each, interspersed with three cycles of ischemia (occlusion of the abdominal aorta artery by atraumatic vascular clamp), also lasting two minutes each.

In group C, the position at the table of the rats, laparotomy of four centimeters, dissection and identification of the artery abdominal aorta were performed. The atraumatic vascular clamp was positioned for a few seconds in the artery but not applied.

**Fig. 3:**
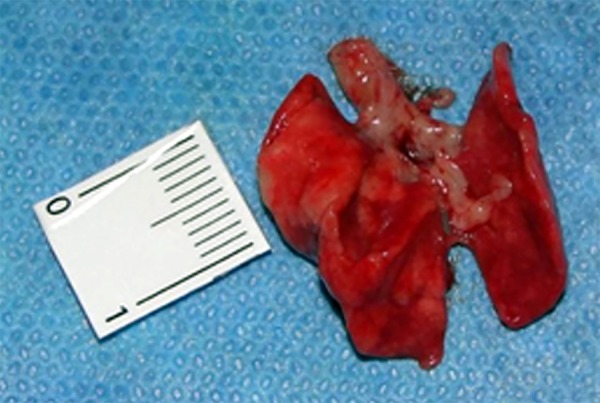
Dried lungs.

Immediately at the end of the reperfusion phase in groups A and B, the abdominal wall was opened again by removing the suture and the lungs were excised (Figure 3), washed with saline 0.9% solution and placed in 10% formaldehyde for later histological analysis.

In all three groups, the animals were euthanized by overdose of those same anesthetic intramuscularly into the right hind limb (100 mg/kg).

**Fig. 4:**
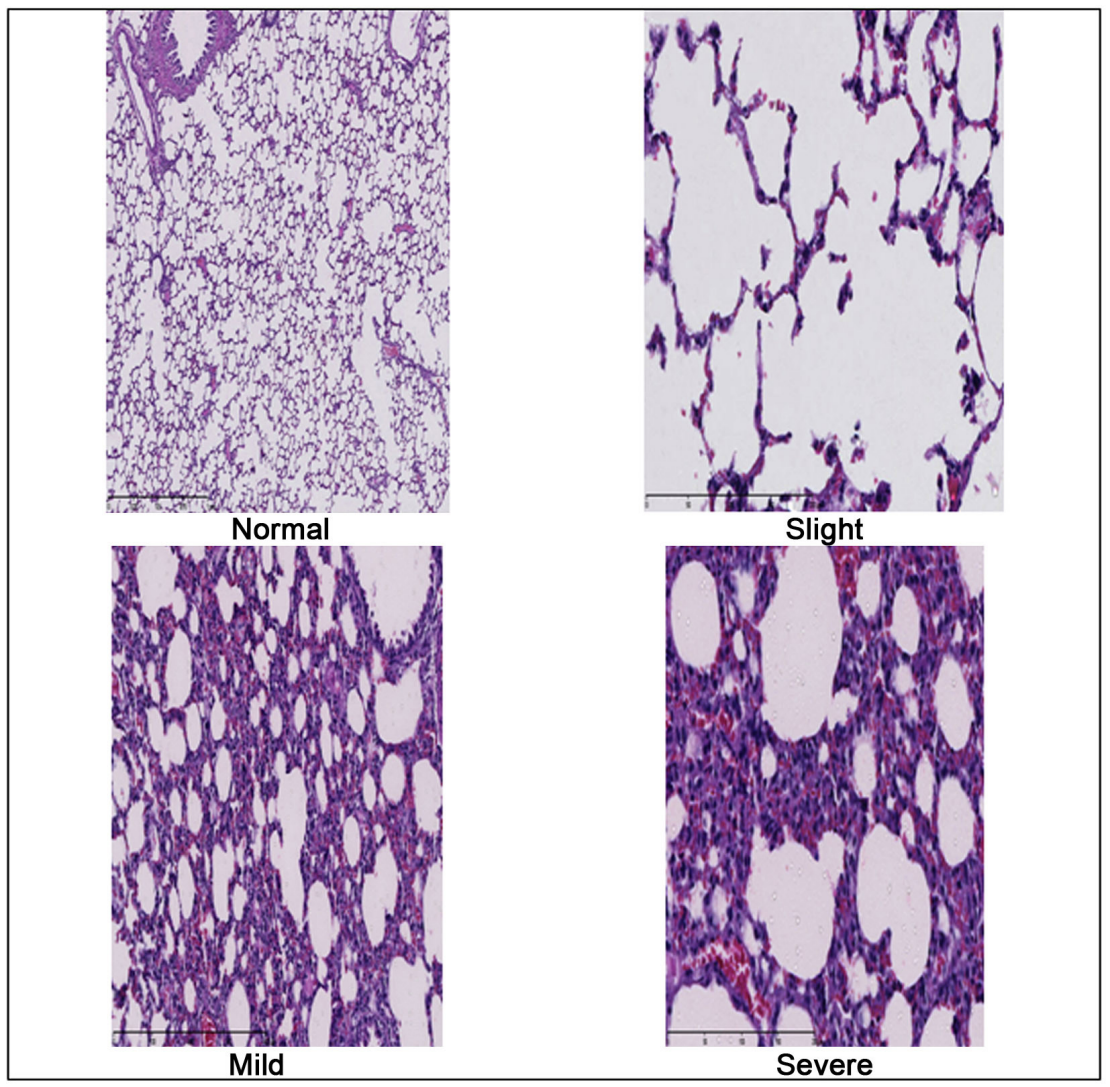
Photograph of histological changes of the lung parenchyma at regular, mild, moderate and intense degrees according Greca et al.^[8]^ classification. (40X optical microscopy - hematoxylin-eosin staining).

For histological analysis of the lungs we used the hematoxylin-eosin and the reading of the slides was performed by the same pathologist, who was blinded to the study, which considered for description of morphological findings, the classification according to the degree of tissue injury described by Greca et al.^[8]^ (Table 1 and Figure 4). We selected three random locations on each lung for analysis: apex, middle third and base, being observed five fields of each slides, resulting in the predominant lesion.

**Table 1:** Classification described by Greca et al.^[8]^.

Grade 1	Grade 2	Grade 3	Grade 4
Normal	Mild	Moderate	Severe
Normal parenchyma on optical microscopy	Focal edema in few alveolar septa, mild congestion, neutrophils in alveolar septa less than 50 per highpower field	Moderate edema in the alveolar septa or mild edema in several septa, moderate congestion, neutrophils in alveolar septa between 50 and 100 per high-power field	Severe edema in the alveolar septa or mild edema in several septa, moderate congestion, neutrophils in alveolar septa more than 100 per high-power field

The values obtained during the study were compared using the Kruskal Wallis test followed by Student-Newman-Keuls. The significance level was 5%. We used the statistical software BioStat version 5.0.

## RESULTS

The search program was accomplished using 24 rats. The results of the histological analysis are shown in Table 2.

**Table 2:** Number of rats according to the focal edema scores at histological examination in groups: A (ischemia and reperfusion), B (remote post conditioning) and C (control).

Focal edema	A	B	C
1 (normal)	0	6	8
2 (slight)	0	2	0
3 (mild)	3	0	0
4 (severe)	5	0	0
Total of animals	8	8	8
Average scores ± standard deviation	^A^3.6±0.5	^B^1.3±0.5	^B^1.0±0.0
Median of scores	4	1	1

Note: Kruskal Wallis test followed by Student-Newman - Keuls test. Equal letters indicate statistically significant difference. Different letters indicate statistically significant difference.

A x B x C: P-value=<0.001

A x B: P-value=0.002

A x C: P-value=<0.001

B x C: P-value=0.572

After histological examination of the degree of inflammation of the lung parenchyma, it was observed that the group C (control) had grade 1 rating according Greca et al.^[8]^ in the eight mice. In Group A (ischemia and reperfusion - I/R) were observed three rats with classification grade 3 and 5 rats with classification grade 4.

In group B (remote ischemic post-conditioning - R-IPosC) were observed six rats with classification grade 1 and two rats with classification grade 2.

**Fig. 5:**
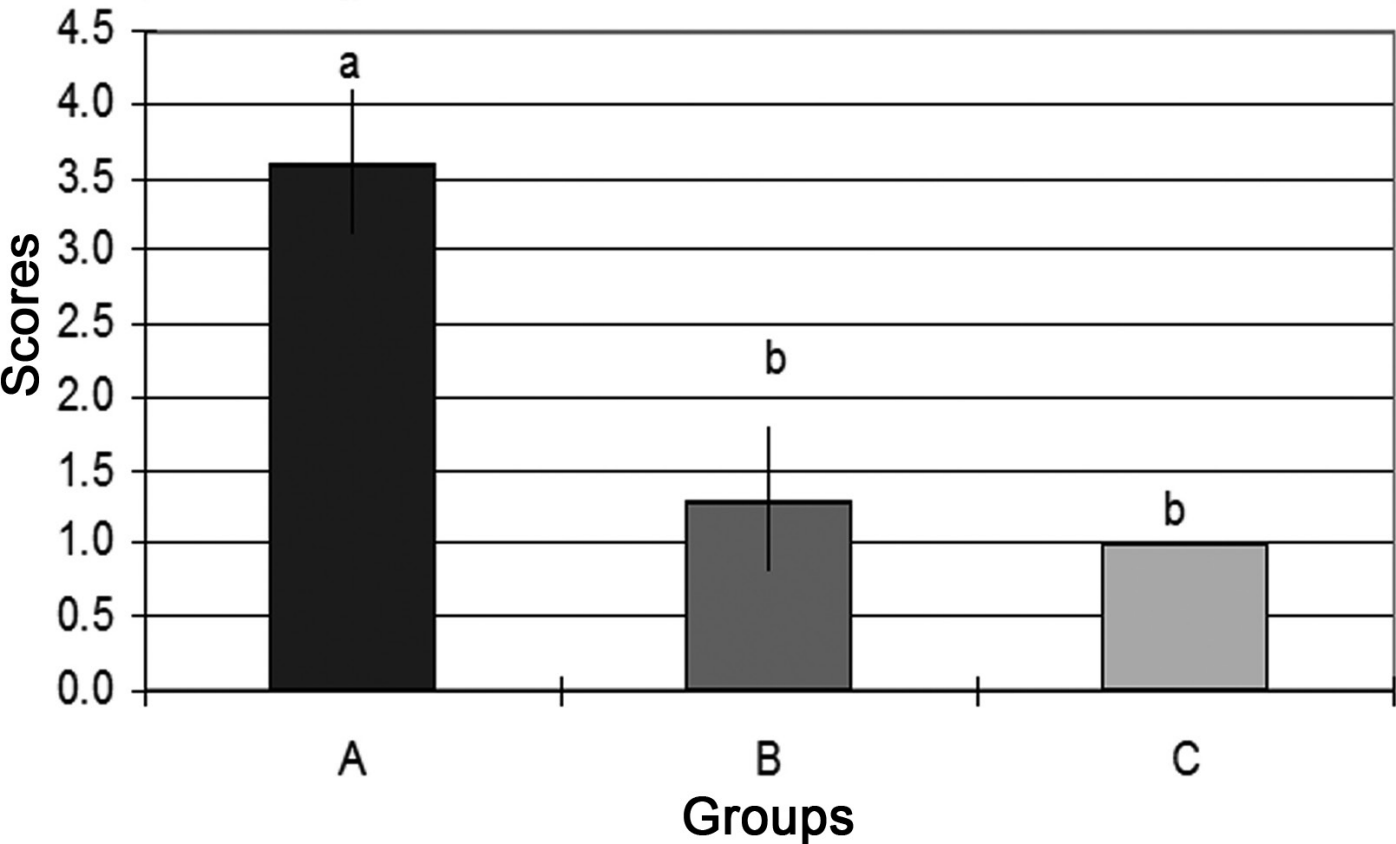
Mean and standard deviation scores of the focal edema at histological examination in groups: A (ischemia and reperfusion), B (remote postconditioning) and C (control). Note: Kruskal Wallis followed by Student-Newman – Keuls test. Equal lowercase letters indicate statistically significant difference. Different lowercase letters indicate statistically significant difference. P-value=<0.001; A x B: P-value=0.002; A x C: P-value=<0.001; B x C: P-value=0.572.

The mean degree of histological classification of Greca et al.^[8]^ of groups I/R and R-PosC were subjected to statistical analysis by Kruskal Wallis test where it was found a *P* value of 0.002 showing that the difference between groups was statistically significant (Figure 5).

## DISCUSSION

According to Pinheiro et al.^[9]^ the main mediators and effectors involved in the PosC mechanisms are: adenosine, nitric oxide, the K ATP-dependent channel, the pro-survival kinase and the mitochondrial permeability transition pore.

The biological expression of these mediators seems to depend on the time of reperfusion/ischemia and animal species. Therefore, it has been suggested that the duration of ischemia and reperfusion protocol in PosC is species dependent and the number of cycles of ischemia and reperfusion seems to be less important than its duration^[9]^. Thus, we observed in this study that with three cycles of two minutes interspersed of ischemia and reperfusion in rats we obtained cellular protection similar to other literature studies with different cycles and times, but always brief periods, non-lethal, of ischemia and reperfusion applied in an organ or tissue.

According Minamino^[10]^ transient limb ischemia is a simple non-invasive stimulation with significant clinical potential and high performance. In addition, the R-PosC procedure can be applied before or during sustained ischemia and/or during reperfusion. The R-PosC could be the most effective way to protect a noble organ such as the heart, brain, lungs and kidneys without applying the method directly on them. It is still uncertain how the R-PosC exerts cardioprotection. However, two main hypotheses are proposed. The neural hypothesis suggests that autocoid released from remote ischemic organ influence the afferent neural pathway, which in turn activates the neural efferent pathways to trigger organ protection. According humoral hypothesis, autacoid released from the remote ischemic tissue are transported to the end-organ, resulting in the activation of kinase signaling pathways in the end body^[10]^.

Loukogeorgakis et al.^[11]^ were the first to assess the effect of R-PosC in humans, demonstrating that it may be induced by limb transient ischemia. The protection offered by the R-PosC was assigned in this research to the activation of K_ATP_ channels.

This study demonstrated that the R-PosC has the ability to mitigate reperfusion injury distant from the model used. Thus, it opens up numerous possibilities of research to study the lung parenchyma protection in the microcirculation stress situations: shock, cardiopulmonary bypass, organ transplantation, acute organ ischemia, compartment syndrome and sepsis.

Considering that the PrC has a protective effect similar to PoC, as demonstrated by Santos et al.^[12]^ in an experimental study of mesenteric ischemia and reperfusion, one would assume that the remote preconditioning (R-PrC) also offers protection similar to R-PosC. However, when confirming this hypothesis, a number of advantages favor the latter, since the most frequent clinical situation is that of establishing the treatment when the process of ischemia has already been occurred and not otherwise.

When confirming the effectiveness of the R-PosC in humans in a manner similar to that observed in the present study, it is believed that there will be great importance in clinical practice, like provoking ischemia and reperfusion cycles in a lower limb, in order to protect the heart in the presence of many conditions such as acute myocardial infarction, shock, pulmonary embolism, etc.

Thus, this research opens new questions that allow extensive research in order to seek ways to promote cellular protection, using the R-PosC, since it has been shown important protection to the lung parenchyma of rats subjected to ischemia and systemic reperfusion process.

## CONCLUSION

The remote ischemic postconditioning was able to minimize the inflammatory lesions of the lung parenchyma of rats subjected to ischemia and systemic reperfusion process.

**Table:** 

**Authors’ roles & responsibilities**	
RCD	Writing; bibliographic survey
JCDVP	Review
ACBA	Guidance
GVRS	Bibliographic survey
RAB	Review
CHMS	Guidance
ERCP	Statistical analysis
JASG	Experimental Surgery
